# Supplementary material for: Development of a computer-aided design software for the quantitative evaluation of aesthetic damage
Source: PLoS One. 2019 Dec 18;14(12):e0226322. doi: 10.1371/journal.pone.0226322 (PMC6919621; doi:10.1371/journal.pone.0226322)
Supplement: S1 Code site — 1. (ZIP) [file pone.0226322.s001.zip › [EESC_jr][Projeto]Relatorio_07_21/reports/templates_zip/gentelella-master.zip!/gentelella-master/production/login.html]

Gentellela Alela! | 


# Login Form

Log in
Lost your password?

New to site?
 Create Account

  

# Gentelella Alela!

©2016 All Rights Reserved. Gentelella Alela! is a Bootstrap 3 template. Privacy and Terms

# Create Account

Submit

Already a member ?
 Log in

  

# Gentelella Alela!

©2016 All Rights Reserved. Gentelella Alela! is a Bootstrap 3 template. Privacy and Terms
